# Early memory loss and financial capacity A “Real World” Assessment

**DOI:** 10.1002/alz.084659

**Published:** 2025-01-03

**Authors:** Peter A Lichtenberg

**Affiliations:** ^1^ Wayne State University, Detroit, MI, USA; Wayne State University/MADRC, Detroit, MI USA

## Abstract

**Background:**

Assessing financial capacity in older adults with memory loss is becoming an increasingly important area so as to minimize wealth loss and cases of financial exploitation. The National Academy of Sciences has recommended “real world” assessment of financial management and yet this continues to be lacking. The WALLET (Wealth Accumulation and Losses in Late‐life Early Cognitive Transitions) study provides a new “real world” approach to assessing financial management.

**Method:**

150 older adults, approximately half with early memory loss and half without, provided 12 months of their checking account statements. These were reviewed for key indicators and then interviews were conducted to insure the older person was the primary manager of the account, to establish annual income and document inflows and outflows. Measures of financial decision‐making, financial literacy, numeracy, memory functioning and indicators for a new Financial Vulnerability Index were collected. Bivariate and multivariate analyses were used to predict excess spending.

**Result:**

Participants with early memory loss reported significantly lower IADL functioning, had lower memory scores (RAVLT), higher financial decision‐making vulnerability scores and higher excess spending than did those with no memory loss. The association between cognitive status and excess spending was explained by adjustment for the financial decision‐making tool (LFDRS) and the new Financial Vulnerability Index (FVI).

**Conclusion:**

Real world assessment of financial functioning in older adults with early memory loss can assist in identifying deficits in financial management. A new method for this assessment was established.

